# Physical Activity, Fitness, and Health-Related Quality of Life in Children and Adolescent Cancer Survivors: A Cross-Sectional Study (iBoneFIT Project)

**DOI:** 10.3390/cancers17061030

**Published:** 2025-03-19

**Authors:** Andrés Redondo-Tébar, Andrea Rodriguez-Solana, Luis Gracia-Marco, Andres Marmol-Perez, José J. Gil-Cosano, Cristina Cadenas-Sánchez, Francisco J. Llorente-Cantarero, Juan Francisco Pascual-Gázquez, María Herrada-Robles, Mairena Sánchez-López, Esther Ubago-Guisado

**Affiliations:** 1 Department of Physical Education and Sports, Faculty of Sport Sciences, Sport and Health University Research Institute (iMUDS), University of Granada, 18071 Granada, Spain; andres.redondo@uclm.es (A.R.-T.); andrearoso@ugr.es (A.R.-S.); amarmol@ugr.es (A.M.-P.); jjgil@uloyola.es (J.J.G.-C.); cadenas@ugr.es (C.C.-S.); mherrada@ugr.es (M.H.-R.); estherug@ugr.es (E.U.-G.); 2 Centro de Estudios Sociosanitarios (CESS), Universidad de Castilla-La Mancha, 16071 Cuenca, Spain; mairena.sanchez@uclm.es; 3 Instituto de Investigación Biosanitaria ibs-Granada, 18012 Granada, Spain; 4 Centro de Investigación Biomédica en Red Fisiopatología de la Obesidad y Nutrición (CIBERobn), Instituto de Salud Carlos III, 28029 Madrid, Spain; fllorente@uco.es; 5 Department of Biological and Health Sciences, Faculty of Health Sciences, Universidad Loyola Andalucía, 41704 Sevilla, Spain; 6 Department of Cardiology, Stanford University, Stanford, CA 94305, USA; 7 Veterans Affair Palo Alto Health Care System, Palo Alto, CA 93404, USA; 8 Instituto de Investigación Biomédica Maimonides (IMIBIC), Hospital Reina Sofía de Córdoba, Universidad de Córdoba, 14006 Córdoba, Spain; 9 Departamento de Didácticas Específicas, Facultad de Educación y Psicología, Universidad de Córdoba, 14006 Córdoba, Spain; 10 Servicio de Onco-Hematología Infantil, Hospital Universitario Virgen de las Nieves, 18013 Granada, Spain; juanf.pascual.sspa@juntadeandalucia.es; 11 Facultad de Educación de Ciudad Real, Universidad de Castilla-La Mancha, 13071 Ciudad Real, Spain

**Keywords:** quality of life, exercise, physical fitness, child, adolescent, cancer survivors

## Abstract

**Background/Objectives**: This study aims to evaluate the health-related quality of life (HRQoL) of children and adolescent cancer survivors in relation to previously published normative values for typically developing children and adolescents, as well as to analyze the differences in HRQoL based on their levels of physical activity and fitness. **Methods**: Cross-sectional study with 116 cancer survivors (12.1 ± 3.3 years, 57.8% boys) from two pediatric oncology units in Andalusia (Spain). HRQoL was assessed using PedsQL 4.0 Generic Core Scales. Physical activity was measured with accelerometers, and fitness was evaluated using self-reported and objective tests for muscular fitness. Independent samples *t*-tests to compare HRQoL between our sample and the normative values published for typically developing children and adolescents of the same age and analysis of covariance (ANCOVA) were conducted to assess differences in HRQoL according to physical activity and fitness categories in our sample. **Results**: Children and adolescent cancer survivors had lower HRQoL scores compared to typically developing children’s and adolescents’ normative values, except for social functioning. Higher levels of moderate-to-vigorous physical activity were associated with better total, physical, and psychosocial HRQoL scores. Children and adolescent cancer survivors with better levels of cardiorespiratory fitness, motor fitness, and flexibility reported better HRQoL scores in total and psychosocial domains. However, muscular fitness (self-reported and objectively measured) did not show a significant difference in HRQoL. **Conclusions**: Children and adolescent cancer survivors experience lower HRQoL than their typically developing counterparts. Engaging in at least 30 min of moderate-to-vigorous physical activity per day is associated with fewer HRQoL impairments. Improved fitness, particularly cardiorespiratory fitness, motor fitness, and flexibility, are associated with better HRQoL outcomes. These findings highlight the association between physical activity, fitness levels, and HRQoL in children and adolescent cancer survivors, suggesting the potential benefits of promoting physical activity and enhancing fitness levels.

## Simple Summary

Childhood cancer survivors often face challenges in maintaining a quality of life comparable to their peers. This study explores how physical activity and fitness levels are associated with health-related quality of life (HRQoL) among young cancer survivors. We found that survivors who engage in daily moderate-to-vigorous physical activity and have higher levels of cardiorespiratory fitness, motor fitness, and flexibility report better HRQoL. These findings indicate an association between physical activity, fitness, and the well-being of pediatric cancer survivors, suggesting that encouraging physical activity may support their HRQoL and help narrow the gap with their peers.

## 1. Introduction

*Cancer* in children and adolescents is a significant global health issue, with varying incidence rates depending on the type of cancer and region. Notable advances in treatment have improved survival rates up to 85% and stabilized the incidence around 13%, with around 300,000 new cases of cancer diagnosed each year in children aged 0–19, making it the second leading cause of death among children and adolescents [1,2,3]. Moreover, the therapeutic interventions employed in the management of pediatric cancer can have detrimental biopsychosocial implications that manifest in the short, medium, or long term and, in some cases, even result in chronic late effects [4,5]. Numerous studies have found the adverse impact of cancer and its treatments on all dimensions of health-related quality of life (HRQoL), with the most significant repercussions on physical and mental health [4,6,7]. Conceptually, HRQoL encompasses a multidimensional construct covering the physical, mental, emotional, social, and behavioral components of well-being [8,9]. Therefore, understanding and preserving the HRQoL of children and adolescent cancer survivors is relevant as it serves as a critical outcome providing scientific evidence for healthcare prevention, health promotion, and clinical decision-making [10,11].

Children and adolescent cancer survivors face long-term health challenges that affect their daily lives, resulting in lower levels of physical activity, reduced fitness, and diminished HRQoL, compared to individuals without a history of cancer [12,13,14]. In this regard, regular participation in physical activity has been shown to enhance fitness among typically developing children and adolescents, encompassing cardiorespiratory, muscular, and motor fitness [15]. Moreover, increasing physical activity and improving fitness in general can reduce the risk of chronic diseases, late effects, and all-cause mortality, among others, making it a valuable tool for children and adolescent cancer survivors to optimize their overall health and HRQoL [16]. However, despite the importance of physical activity and fitness for children and adolescent cancer survivors, it is known that physical activity and fitness levels of children and adolescent cancer survivors tend to decline following a pediatric cancer diagnosis and remain stable after the completion of treatment or years later [12,17,18].

Although research examining the effects of physical activity and fitness is not novel in the general population, it has become a topic of interest in children and adolescent cancer survivors [19]. In this sense, international consensus in pediatric oncology recommends exercise for all children and adolescents at all times (i.e., after diagnosis and during and after treatment) as most of them are not active enough to receive its benefits [20,21,22]. However, the majority of children and adolescent cancer survivors fail to meet the recommended levels of physical activity for typically developing children and adolescents (an average of 60 min moderate-to-vigorous physical activity per day) [23] even after the completion of treatment or years later [12,17,18]. Indeed, it has been postulated that specific recommendations must be established for this population as the recommendations used for typically developing children and adolescents are not ideal for children and adolescent cancer survivors [19,24,25] who may not reap the same rewards from physical activity as their typically developing peers. What is more, studies that investigate the association between different fitness components, physical activity, and HRQoL among children and adolescent cancer survivors are limited.

Therefore, the aims of this study were i) to describe the HRQoL of children and adolescent cancer survivors compared to previously published normative values for typically developing children and adolescents and ii) to analyze the differences in HRQoL according to physical activity and fitness levels in children and adolescent cancer survivors.

## 2. Materials and Methods

### 2.1. Study Design and Participants

This study was a cross-sectional analysis of baseline data measurements from the iBoneFit cluster-randomized controlled trial (isrctn.com, ISRCTN61195625) conducted between October 2020 and March 2022. The main objective of iBoneFIT was to assess the impact of a physical activity intervention program on body composition, especially bone health, in children and adolescents who had survived pediatric cancer. Participants were eligible if they were aged 6–18 years, had been diagnosed with cancer at least one year before enrolment, were free from active cancer treatment, and had prior exposure to radiotherapy and/or chemotherapy [26]. However, individuals who met certain exclusion criteria were not included in the study. Participants were excluded if they were concurrently enrolled in another study that could pose additional risks, cause discomfort, or influence the results of either study. Individuals with a prior diagnosis of anorexia nervosa or bulimia, known pregnancy, or a history of alcohol or drug abuse were also excluded. Furthermore, children requiring chronic oral glucocorticoid therapy, those with an injury that could impair daily activities and be exacerbated by exercise, and those with a lower limb prosthesis preventing accurate bone assessment were not eligible for participation.

Finally, 116 participants aged 6–18 were recruited from the pediatric oncology and hematology units of two provincial university reference hospitals in Spain (“Virgen de las Nieves” [Granada] and “Reina Sofia” [Cordoba]). Although 116 children and adolescent cancer survivors were recruited for the study, it is important to acknowledge that the sample size may vary slightly for certain variables due to missing data (i.e., some participants were unable to perform some of the tests or declined a particular test during their assessment). Written informed consent for participation was obtained from the children’s parents, which could be revoked by either the parents or the child or adolescent. Trained investigators measured all variables to minimize interobserver variability. The study protocol was approved by the Ethics Committee on Human Research of the Regional Government of Andalusia (R4500).

### 2.2. Fitness

Fitness was assessed using the self-reported Spanish version of the 5-item International Fitness Scale (IFIS) which has been validated for children and adolescents aged 9–17.5 years [27,28]. Although the entire sample completed the questionnaire, children aged 6–8 years received assistance from their parents to respond appropriately. The questions asked about perceived fitness, with responses reflecting participants’ perception of their fitness level compared to their peers. The IFIS has five dimensions: overall fitness, cardiorespiratory fitness, muscular fitness, motor fitness, and flexibility. It includes 5 possible answers (i.e., very bad, bad, acceptable, good, and very good). The IFIS scoring system is based on the sum of responses across all five dimensions, with higher total scores indicating better perceived fitness. In addition, upper and lower body muscular fitness was objectively assessed using the handgrip strength (TKK 5101 Grip D, Takey, Tokyo, Japan) and standing long jump tests, respectively, included in the Alpha-Fitness Test Battery [29]. Both tests were performed twice during the evaluation, and the best score was retained. The participants were grouped into three fitness categories according to the test: “*very poor-poor fitness*”, which included individuals with fitness levels falling below or equal to the 10th percentile on the IFIS or performing below the 10th percentile on handgrip or standing long jump tests; “*average fitness*”, including those within the 10th to 30th percentile on the IFIS or scoring between 10th and 30th percentile on handgrip or standing long jump tests; and “good-very good fitness”, which included participants with fitness at or above 30th percentile on the IFIS or scoring at or above 30th percentile on handgrip or standing long jump tests [27].

### 2.3. Physical Activity

Physical activity was assessed using the wrist-worn tri-axial ActiGraph wGT3x-BT accelerometer (ActiGraph GT3X, Pensacola, FL, USA). Data was collected over 8 consecutive days (24 h each). This protocol ensured the inclusion of both weekdays and weekends. The accelerometer recorded sedentary behavior, light physical activity, and moderate-to-vigorous physical activity, and was only removed for water-based activities. A day was considered valid if the device registered at least 23 h of wear time as participants wore the accelerometer both during the day and at night [30]. Survivors with at least one valid day were included in the analysis, with sensitivity analyses showing similar results when compared to those participants with at least three valid weekdays and one weekend day. The accelerometers started at a sampling frequency of 90 Hz. Raw data were processed using the open-source R package GGIR (v.2.1–3), which calculated variables for weekdays and weekends [31]. Appropriate thresholds were then used to distinguish between the various activity levels (35 mg for sedentary behavior, 35–200 mg for light physical activity, and 200 mg for moderate-to-vigorous physical activity) [32]. Given the lack of specific guidelines for children and adolescent cancer survivors and considering that the World Health Organization’s recommendation of 60 min of at least moderate intensity physical activity used for typically developing children and adolescents may not be ideal [12,17,18,19,24,25], the 60 min reference value was thoughtfully divided to establish cutoff points above and below it, taking into consideration the unique characteristics of the participants in the study. Consequently, the participants were categorized into three groups based on their daily moderate-to-vigorous physical activity: those who practiced up to 30 min, between 30 and 60 min, and more than 60 min.

### 2.4. Health-Related Quality of Life

Health-related quality of life was assessed using the age-appropriate Spanish version of the 23-item, self-reported questionnaire for children and adolescents aged 5–18 years, known as PedsQL 4.0 Generic Core Scales [33]. The questionnaire utilizes a 5-Likert scale (0 = never a problem; 1 = almost never a problem; 2 = sometimes a problem; 3 = often a problem; 4 = always a problem) to assess the extent to which each item has affected the participants over the last month. PedsQL 4.0 covers four subscales: physical (8 items), emotional (5 items), social (5 items), and school (5 items). The item scores were reverse scored and linearly transformed to a 0–100 scale (0 = 100, 1 = 75, 2 = 50, 3 = 25, and 4 = 0). The scale scores were computed as the sum of the items answered divided by the number of items. However, if more than 50% of items within a scale were missing, the scale score was not calculated. Therefore, PedsQL 4.0, which allows for obtaining a total health summary score (based on the physical and the psychosocial functioning subscales, comprising 23 items), a physical health summary score (based on the physical functioning subscale, comprising 8 items), and a psychosocial health summary score (based on the emotional, the social, and the school functioning subscales, comprising 15 items) were used, with higher scores indicating a higher HRQoL. Additionally, normative scores from the PedsQL 4.0 [34] were used to compare children and adolescent cancer survivors in our sample with the published normative values for typically developing children and adolescents of the same age. Furthermore, PedsQL 4.0 cutoff point scores from the child self-reporting [34] were used to determine the at-risk status of impaired HRQoL for each dimension (total health summary score = 69.71, physical health summary score = 72.98, and psychosocial health summary score = 66.03 [emotional =59.57, social =66.61, school =62.99]).

### 2.5. Covariates

Height (cm) was assessed using a precision stadiometer (SECA 225; Hamburg, Germany) to the nearest 0.1 cm. Weight (kg) was measured using an electronic scale (SECA 861; Hamburg, Germany) with precision of 0.1 kg. Body mass index was calculated as weight (kg)/height (m^2^). The peak height velocity (PHV) is a benchmark for somatic development, representing the period of maximal stature growth in children and adolescents. The PHV offset was determined for each participant by validated algorithms specific to their sex and sitting height [35]. Clinical data were also used to determine the type of neoplasm and length of time (in years) between diagnosis and testing.

### 2.6. Statistical Analysis

Descriptive characteristics of the study sample were calculated as means and standard deviations or percentages for continuous and categorical variables, respectively. The normal distribution of all variables was evaluated using graphical procedures and the Shapiro–Wilk test. Cronbach’s alpha was calculated for the six PedsQL 4.0 scales, and the five IFIS scales showed excellent (0.9) and high (0.8) internal consistency, respectively [36].

Differences in HRQoL between the self-reports of our sample and the previously published normative values from their same-aged typically developing children and adolescents [34] were tested using independent samples *t*-test analysis. The average age of the typically developing children and adolescents was 9.8 years (SD = 3.15) for 3139 boys (52.4%) and 2852 girls (47.6%), ranging from 5.0 to 16.4 years.

Moreover, an analysis of covariance (ANCOVA) with Bonferroni post hoc was used to assess differences in HRQoL according to physical activity and fitness categories, considering potential factors as covariates that may influence the HRQoL of children and adolescents, such as sex, body mass index, PHV offset, and years from diagnosis to testing [37]. Participants in the ‘*average fitness*’ category were excluded to ensure a clearer distinction between lower and higher fitness levels. Cohen’s d effect size for the *t*-test and partial eta squared (*η_p_^2^*) for the ANCOVA test were calculated. Cohen’s d and partial eta squared (*η_p_^2^*) indicate small effect (≥0.2–0.5; ≥0.01–0.06, respectively), intermediate effect (≥0.5–0.8; ≥0.06–0.14, respectively) and strong effect (≥0.8; ≥0.14, respectively) sizes [38].

Statistical analyses were performed using IBM SPSS Statistics Statistical v28 (IBM Corp., Armonk, NY, USA). Statistical significance was set at *p* < 0.05.

## 3. Results

### 3.1. Participants’ Characteristics

Table 1 summarizes the descriptive characteristics of the 116 pediatric cancer survivors included in the study, with a mean age of 12.1 years (SD = 3.3). Of these, 67 (57.8%) were boys and 49 (42.2%) were girls. Regarding cancer type, 70 participants (60.9%) had hematologic tumors, while 45 (39.1%) had solid tumors.

### 3.2. Physical Activity

**Figure 1:**
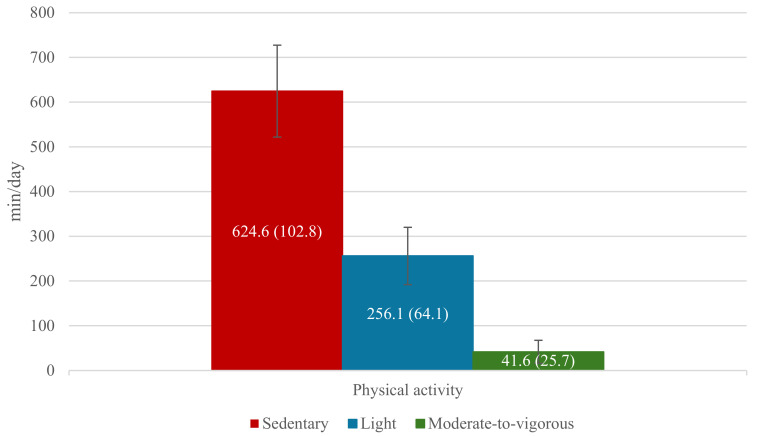
Daily time spent in sedentary, light, and moderate-to-vigorous physical activities of the study sample (n = 110).

Figure 1 illustrates the distribution of time spent on different physical activity intensities among 110 participants, with an average number of valid days of 7.45 (SD = 0.79) and a range of 2 to 8 days. On average, children and adolescents spent a substantial portion of their day—624.6 min (SD = 102.8)—engaged in sedentary activities. Time spent in light physical activity accounted for 256.1 min per day (SD = 64.1), whereas moderate-to-vigorous physical activity represented only 41.6 min per day (SD = 25.7). These findings highlight the predominance of sedentary behaviors within this population.

### 3.3. Fitness

**Figure 2:**
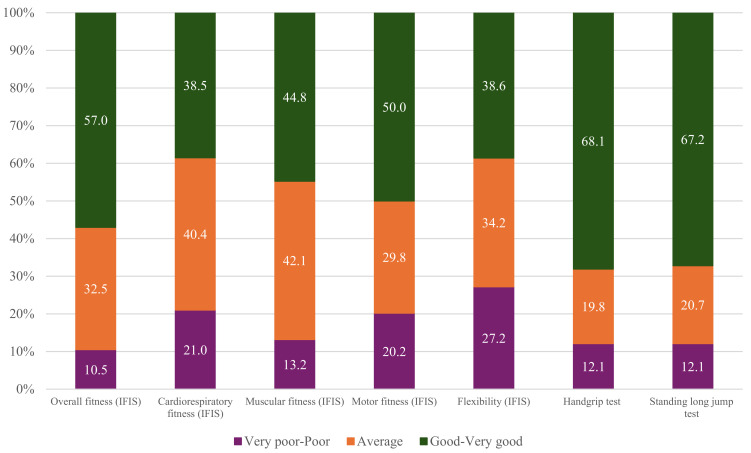
Self-reported (International Fitness Scale [IFIS]; n = 114) and objective fitness (handgrip and standing long jump tests; n = 116 and n = 115, respectively) levels of the study sample. Data are presented as accumulated percentages. The categories of fitness correspond to percentile ≤ 10, ‘*Very poor-Poor*’; percentile 10–30, ‘*Average*’; and percentile ≥ 30, ‘*Good-Very good*’.

Self-reported fitness, assessed through the IFIS, was available for 114 participants, while objective fitness measurements were obtained for all 116 participants using the handgrip strength test and the standing long jump test (Figure 2).

In the handgrip test, grip strength values varied significantly across fitness categories: participants classified as having very-poor-to-poor fitness had a mean grip strength of 7.7 kg (SD = 0.8), those in the average fitness category showed a mean of 10.8 kg (SD = 1.1), and those with good-to-very-good fitness exhibited a mean grip strength of 22.0 kg (SD = 7.6). A similar pattern was observed in the standing long jump test where jump distances increased from 67.3 cm (SD = 12.2) in the very-poor-to-poor category, to 93.9 cm (SD = 6.2) in the average category, and up to 134.3 cm (SD = 26.0) in the good-to-very-good fitness group.

### 3.4. Health-Related Quality of Life (HRQoL)

The HRQoL outcomes of pediatric cancer survivors were compared to previously published normative data [34] from age-matched typically developing children and adolescents (Table 2). Overall, cancer survivors exhibited significantly lower scores in total, physical, and psychosocial health summary measures, including emotional and school functioning, when compared to their typically developing peers (*p* < 0.001; Cohen’s d ≥ 0.63 and ≤ 0.90). However, social functioning scores did not differ significantly between the two groups (*p* > 0.50), suggesting that this domain may be relatively preserved in pediatric cancer survivors.

### 3.5. Association Between Fitness and HRQoL

The relationship between fitness and HRQoL was examined, with mean-adjusted differences reported in Table 3. Children and adolescents who perceived themselves as having good overall fitness, cardiorespiratory fitness, motor fitness, and flexibility scored significantly higher in total and psychosocial health measures, including social functioning, compared to those with lower fitness levels (*p* ≤ 0.044; *η_p_*^2^ ≥ 0.06 and ≤ 0.37).

Furthermore, participants who reported higher cardiorespiratory and motor fitness levels also showed better physical health and school functioning. Specifically, those with higher cardiorespiratory fitness demonstrated better emotional functioning as well (*p* ≤ 0.018; *η_p_*^2^ ≥ 0.08 and ≤ 0.33). However, muscular fitness, whether self-reported or objectively measured, did not show a significant association with any HRQoL scale.

### 3.6. Associations Between Physical Activity and HRQoL

**Figure 3:**
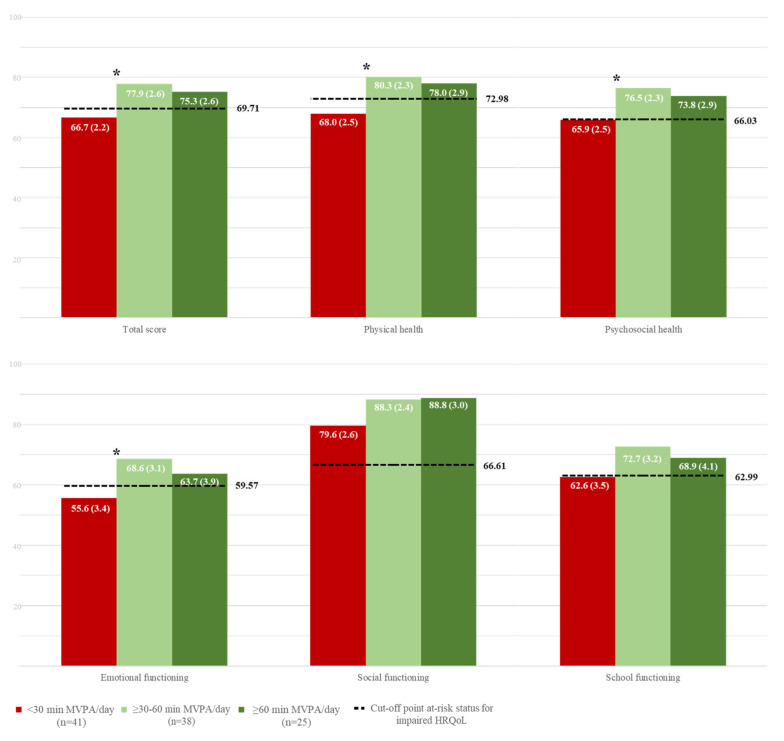
Mean-adjusted differences between moderate and vigorous physical activity (MVPA) categories in health-related quality of life of the study sample. Data are presented as mean-adjusted and standard errors (controlling for sex, body mass index, peak height velocity offset, and time between diagnosis and study evaluation). The categories of MVPA correspond to: <30 min/day, less than 30 min per day; ≥30–60 min/day, between 30 to 60 min per day; and ≥60 min/day, 60 min or more per day. The dotted line represents the cutoff point for at-risk status regarding impaired health-related quality of life. * Indicates statistically significant differences (*p*-values <0.05) between the adjacent categories. Data are presented as accumulated percentages. The categories of fitness correspond to percentile ≤10, ‘*Very poor-Poor*’; percentile 10–30, ‘*Average*’; and percentile ≥30, ‘*Good-Very good*’.

Figure 3 presents the relationship between MVPA and HRQoL in pediatric cancer survivors. Engaging in at least 30–60 min of MVPA per day was associated with significantly higher total, physical, and psychosocial health scores, including emotional and social functioning, compared to those who engaged in less than 30 min per day (*p* ≤ 0.020; *η_p_*^2^ ≥ 0.05 and ≤ 0.10). Notably, among all HRQoL measures, only the physical health scale showed a significant advantage in those engaging in more than 60 min of MVPA per day compared to those performing less than 30 min (*p* = 0.029; *η_p_*^2^ = 0.77).

## 4. Discussion

The findings of this study suggest the following: (i) Children and adolescent cancer survivors had lower HRQoL scores than their typically developing children and adolescent counterparts, except for social functioning where no differences were observed. (ii) Children and adolescent cancer survivors who engaged in higher levels of daily moderate-to-vigorous physical activity and those with a better perception of fitness showed significantly higher HRQoL. Moreover, (iii) children and adolescents who participated in moderate-to-vigorous physical activity for over 30 min per day had higher HRQoL scores compared to their peers who participated for less than 30 min per day. The findings of this study highlight the association between physical activity, perceived fitness, and overall quality of life in childhood cancer survivors, emphasizing the relevance of promoting an active lifestyle in this population.

Pediatric cancer is a major cause of death and is highly traumatic [39,40]. Due to the unique nature, diverse treatment approaches are employed, and interventions during critical developmental stages can profoundly impact individuals’ health, perspectives, and values [6,41]. Despite advancements in life expectancy, survivors often encounter a wide range of challenges that extend beyond treatment and mere survival of the disease [4,42,43]. These challenges include physical health issues and heightened psychosocial risks when compared to their non-cancer peers, such as pain, fatigue, emotional distress, fear of recurrence, sleep disorders, and social isolation [4,41,42,43,44,45,46,47,48]. It is important to note that these effects frequently have a pervasive impact on various aspects of the lives of children and adolescents diagnosed with cancer [4,6,7,49]. These effects persist from childhood into adulthood, encompassing their physical and psychosocial well-being, leading to a significant decline in their HRQoL [4,6,7,49,50]. However, in social relations, once reintegration into school or high school context is achieved, the impact on their psychosocial needs is minimized, in particular the relationships with friends [51]. Following previous review studies [51,52,53], our results revealed that children and adolescent cancer survivors had lower scores on total, physical, and psychosocial HRQoL scales than their typically developing children and adolescents counterparts, except for social functioning, where no differences were found.

Most studies have found a positive impact of physical activity on HRQoL in children and adolescent cancer survivors [54,55,56,57,58,59,60,61], while other research has suggested a bidirectional relationship between physical activity and HRQoL, indicating a mutual influence between both factors [62]. Indeed, this positive outcome could be attributed to the significant potential for improvement in HRQoL within this specific population. The findings of our study, using accelerometers to quantify physical activity, confirm the trend shown in previous works showing that children and adolescent cancer survivors who spent more time in physical activity had better scores in total, physical, and psychosocial scores of HRQoL [57,58,59,60]. Interestingly, our data show that performing at least 30 min of physical activity per day may be associated with better HRQoL in children and adolescent cancer survivors. This finding could be especially important because children and adolescent cancer survivors are known to be less physically active than their typically developing counterparts and several factors may contribute to the noncompliance with the physical activity recommendations in this population [12,17,18]. These factors include long-term side effects of treatment, such as fatigue and muscular weakness, fear of physical activity due to health concerns, lack of support and guidance in engaging in physical activities, logistical barriers such as limited access to sports facilities, and changes in self-image and confidence following the cancer experience [12,63,64,65]. Addressing these barriers is crucial for promoting physical activity in children, and it is possible to motivate and engage children with cancer in physical activity during and after treatment [66,67,68]. Moreover, although meeting the recommended amount of moderate-to-vigorous physical activity per day can be challenging in this population, engaging in at least 30 min is associated with better HRQoL.

Physical activity has been associated with fitness and health benefits in typically and non-typically developing children [69]. Recent systematic reviews have shown that cancer negatively affects fitness (cardiorespiratory, muscular, motor, and flexibility functions) [19,70,71,72]. Studies conducted on typically developing children and adolescents have shown that higher fitness levels are associated with better physical and psychosocial health indicators [73,74,75,76,77,78,79,80,81]. However, the association between fitness and HRQoL has been less studied in children and adolescent cancer survivors [82]. To the best of our knowledge, cross-sectional studies in children and adolescent cancer survivors have found that better cardiorespiratory [83,84], muscular [85], motor [83] and flexibility [83] fitness were associated with better total [84,85], physical [84,85] and psychosocial [83,84,85] scores of HRQoL. In general, the better the fitness, the better the HRQoL for all forms of cancer examined, at any stage of treatment, and even in the presence of treatment complications [22,57,86,87]. In our study, those who perceived better levels of cardiorespiratory fitness, motor fitness, or flexibility had better total and psychosocial scores. Moreover, those who perceived higher levels of cardiorespiratory and motor fitness presented greater physical scores. Although the participants’ subjective perception and objectively measured muscular fitness may have improved over time, from the point of diagnosis to years after completing their treatment, participants performing better in muscular fitness did not present greater scores in any of the HRQoL scales. Our findings are not in agreement with one previous cross-sectional [85] study which reported better HRQoL scores according to muscular fitness. The discrepancies between our study and the study developed by Deisenroth et al. (2016) [85] may be explained by the fact that their study was carried out in the primary phases of cancer treatment, the phase that most aggressively affects children and adolescents recently diagnosed with cancer at a biopsychosocial level. Currently, our data indicate that 50% and 90% of participants, based on their age and sex, scored below the European average for handgrip and standing long jump tests, respectively. Consequently, the lack of significant findings regarding muscular fitness, both in terms of self-perceived and objectively measured fitness, may be related to muscle mass and strength impairments documented during childhood cancer treatment. These impairments often persist over time in this vulnerable population, and their improvements may not be sufficient to positively impact HRQoL [88].

This study has several strengths and limitations that should be considered. One of its main strengths is the examination of HRQoL in children and adolescent cancer survivors, which is a critical aspect of their overall well-being, extending beyond the treatment period into the post-treatment phase. Comprehensive assessments were conducted using validated measures to evaluate HRQoL, physical activity, and fitness while accounting for relevant covariates. A notable methodological strength is the use of accelerometers to objectively measure physical activity, reducing reliance on self-reported data and minimizing potential bias. Additionally, the inclusion of a relatively large sample of 116 pediatric cancer survivors, compared to previous studies, enhances the generalizability of the findings. However, this study also has limitations. The cross-sectional design precludes the ability to establish causality between physical activity, fitness, and HRQoL. While meaningful associations were observed, longitudinal studies are needed to determine causal relationships. The impact of the COVID-19 pandemic during the study period represents another limitation. Due to pandemic-related restrictions, not all fitness components could be objectively assessed. Although the safety of participants and their families was prioritized, the inability to perform certain tests may have affected the completeness of the fitness evaluation. Moreover, the pandemic itself could have influenced HRQoL in ways that were not fully captured in this study. Regarding the use of normative data, this study utilized the PedsQL values published by Varni et al. (2003) [34] as a reference for typically developing children and adolescents. While these values are widely used, they may not fully represent current normative standards due to generational differences. Additionally, the reference sample was collected over 20 years ago from a predominantly white population in the southern USA, which may limit its comparability with the present study population. The lack of more recent standardized data constrains direct comparisons, and future research should incorporate updated benchmarks for a more accurate interpretation of HRQoL outcomes. Limitations related to fitness assessment should also be considered. The IFIS, while validated for certain age groups (9–17.5 years), lacks explicit validation for children aged 6, 7, and 8 years, who comprised a total of 16 participants in this study. As a result, caution is needed when interpreting the findings in these age groups. Furthermore, pediatric cancer survivors may experience cognitive challenges that affect the accuracy of self-reported fitness levels, potentially impacting the reliability of IFIS responses. Childhood cancer is a rare disease; therefore, the heterogeneity of the study group should be taken into account as a limitation. However, combining different cancer types is a common approach in pediatric cancer research to facilitate more comprehensive analyses. While including children with various oncological diagnoses enhances the real-world applicability of the findings, different cancer types and treatment regimens may impact HRQoL outcomes in distinct ways. Due to the sample size constraints, conducting subgroup analyses based on cancer type was not feasible. Future research should aim for larger and more homogeneous samples to allow for a more detailed exploration of these differences. Additionally, other factors such as socioeconomic status, ethnicity, and social support should be considered in future studies to provide a more comprehensive understanding of HRQoL in this population.

## 5. Conclusions

Our findings indicate that children and adolescent cancer survivors have lower HRQoL scores than typically developing children and adolescents. Moreover, individuals engaging in higher daily moderate-to-vigorous physical activity and having higher levels of cardiorespiratory fitness, motor fitness, and flexibility report better HRQoL than their counterparts. Notably, those who engaged in at least 30 min of moderate-to-vigorous physical activity per day appeared to have a higher likelihood of better HRQoL. These findings emphasize the importance of encouraging physical activity and a positive attitude towards fitness in all relevant contexts as key factors potentially linked to improved HRQoL in children and adolescent cancer survivors. Future policies should prioritize the holistic well-being of childhood cancer survivors, recognizing the interplay between physical activity, fitness, and HRQoL outcomes. By incorporating these findings into future interventions, we can better support the well-being and long-term quality of life of these vulnerable individuals.

## Figures and Tables

**Table 1:** Characteristics of the study sample.

	n	Value
**Age at diagnosis (years; SD)**	116	5.6 (3.4)
* Infant (0–1 years)*	18	0.9 (0.5)
* Toddler (2–4 years)*	26	3.2 (0.6)
* Young child (5–7 years)*	42	5.7 (1.0)
* Child (8–12 years)*	20	10.1 (1.1)
* Adolescent (13–18 years)*	7	14.0 (0.6)
**Time from diagnosis to testing (years; SD)**	110	06.4 (3.9)
**Age at testing (years; SD)**	116	12.1 (3.3)
* Young child (6–7 years)*	16	07.2 (0.5)
* Child (8–12 years)*	49	10.4 (1.5)
* Adolescent (13–18 years)*	51	15.3 (1.5)
**Sex (n, %)**	116	
* Boys*		67; 57.8
* Girls*		49; 42.2
**Type of neoplasm (n, %)**	115	
* Soft tumors (hematologic)*		70; 60.9
* Solid tumors*		45; 39.1
**Distribution of cancer types (n, %)**	116	
* Acute lymphoblastic leukemia*		45; 38.8
* Lymphoma*		14; 12.1
* Central nervous system tumor*		11; 09.5
* Renal tumor*		9; 07.8
* Neuroblastoma*		8; 06.9
* Malignant bone tumor*		8; 06.9
* Histiocytosis*		6; 05.2
* Soft tissue and other extraosseous sarcomas*		5; 04.3
* Others (retinoblastoma, hepatic tumor, other malignant epithelial neoplasms, unknown)*		10; 08.6
**Anthropometry**	116	
* Weight (kg; SD)*		46.6 (18.0)
* Height (cm; SD)*		147.5 (17.1)
* Body mass index (kg/m^2^;SD)*		20.7 (4.7)
* Peak height velocity offset (years; SD)*		−0.8 (2.7)

Data are presented as means and standard deviations, except for sex, type of neoplasm, and distribution of cancer types, which are shown as quantity and percentage.

**Table 2:** Comparison of health-related quality of life domains on the Pediatric Quality of Life InventoryTM 4.0 (PedsQL 4.0) between the self-reports of children and adolescent cancer survivors in our sample and the published normative values of same-aged typically developing children and adolescents.

	n	Children and Adolescent Cancer Survivors	n	Normative Values	*p*-Value	Cohen’s d
**Total score**	112	72.5 (12.2)	5972	82.9 (13.2)	**<0.001**	0.79
**Physical health**	112	74.4 (13.9)	5962	86.9 (13.9)	**<0.001**	0.90
**Psychosocial health**	112	71.5 (13.2)	5963	80.7 (14.7)	**<0.001**	0.63
**Emotional functioning**	112	62.0 (18.0)	5961	78.2 (18.6)	**<0.001**	0.87
**Social functioning**	112	84.9 (13.6)	5948	84.0 (17.4)	0.507	-
**School functioning**	112	67.7 (18.5)	5908	79.9 (16.9)	**<0.001**	0.70

Data are presented as means and standard deviations. Cohen’s d indicates small, medium, or large effect size—respectively, 0.2, 0.5, and 0.8 [38]. *p*-values in bold indicate statistically significant differences (*p* < 0.05) between groups.

**Table 3:** Mean-adjusted differences in health-related quality of life between self-perceived fitness on the International Fitness Scale (IFIS) and objectively measured fitness categories with handgrip and standing long jump tests by the study sample.

	n	Total Score	*p*-Value (*η_p_^2^*)	Physical Health	*p*-Value (*η_p_^2^*)	Psychosocial Health	*p*-Value (*η_p_^2^*)	Emotional Functioning	*p*-Value (*η_p_^2^*)	Social Functioning	*p*-Value (*η_p_^2^*)	School Functioning	*p*-Value (*η_p_^2^*)
**IFIS-Overall fitness**													
Very poor to poor	12	63.8 (4.3)		66.1 (4.9)		62.6 (4.5)		58.8 (6.3)		73.4 (4.2)		55.6 (6.6)	
Good to very good	63	74.4 (1.6)	**0.030** **(0.07)**	76.0 (1.9)	0.077	73.5 (1.7)	**0.035** **(0.06)**	63.5 (2.4)	0.515	88.1 (1.6)	**0.003** **(0.12)**	69.0 (2.5)	0.071
**IFIS-Cardiorespiratory fitness**													
Very poor to poor	23	61.3 (2.4)		61.1 (2.7)		61.4 (2.6)		54.1 (3.8)		75.1 (3.1)		54.9 (3.8)	
Good to very good	41	80.0 (1.7)	**<0.001 (0.37)**	80.5 (1.9)	**<0.001 (0.33)**	79.7 (1.8)	**<0.001 (0.33)**	69.6 (2.7)	**0.003** **(0.14)**	92.7 (2.2)	**<0.001 (0.25)**	76.6 (2.7)	**<0.001 (0.25)**
**IFIS-Muscular fitness**													
Very poor to poor	14	67.7 (3.4)		70.2 (4.0)		63.4 (3.6)		53.9 (5.5)		83.2 (3.3)		62.2 (4.8)	
Good to very good	50	74.7 (1.7)	0.083	76.7 (2.0)	0.167	73.6 (1.8)	0.090	64.7 (2.8)	0.092	88.5 (1.7)	0.170	67.6 (2.4)	0.331
**IFIS-Motor fitness**													
Very poor to poor	23	65.8 (2.8)		66.6 (3.0)		65.4 (3.1)		58.5 (4.5)		77.5 (3.3)		60.2 (4.2)	
Good to very good	54	77.2 (1.7)	**0.001** **(0.13)**	79.5 (1.8)	**<0.001 (0.15)**	76.0 (1.9)	**0.008** **(0.09)**	66.2 (2.7)	0.172	88.8 (2.0)	**0.008** **(0.10)**	72.9 (2.6)	**0.018** **(0.08)**
**IFIS-Flexibility**													
Very poor to poor	31	67.9 (2.3)		69.3 (2.6)		67.1 (2.6)		59.4 (3.4)		78.8 (2.4)		63.0 (3.7)	
Good to very good	42	74.9 (2.0)	**0.029** **(0.07)**	76.2 (2.2)	0.055	74.2 (2.2)	**0.044** **(0.06)**	64.6 (2.9)	0.255	88.2 (2.0)	**0.005** **(0.11)**	69.7 (3.1)	0.185
**Handgrip test**													
Very poor to poor	13	67.1 (4.3)		68.2 (4.6)		66.5 (4.7)		60.0 (6.0)		78.5 (5.0)		61.1 (6.9)	
Good to very good	74	73.2 (1.5)	0.206	76.4 (1.6)	0.122	71.5 (1.7)	0.340	60.7 (2.1)	0.915	84.9 (1.8)	0.251	69.0 (2.4)	0.302
**Standing long jump test**													
Very poor to poor	12	69.8 (3.5)		69.8 (4.0)		69.7 (3.8)		68.1 (5.3)		77.2 (4.3)		63.8 (5.2)	
Good to very good	72	74.5 (1.4)	0.221	77.3 (1.6)	0.092	73.0 (1.5)	0.431	63.2 (2.1)	0.411	86.9 (1.7)	0.051	69.0 (2.0)	0.363

Data are presented as mean-adjusted and standard errors (controlling for sex, body mass index, peak height velocity offset, and time between diagnosis and study evaluation). The categories of fitness correspond to percentile ≤10, ‘*Very poor-Poor*’ and percentile ≥P30, ‘*Good-Very good*’. *p*-values in bold indicate statistically significant differences (*p* < *0*.05) between categories.

## Data Availability

Data supporting the findings of this study are available upon request from the corresponding author.
